# Regional characterisation of TRPV1 and TRPA1 signalling in the mouse colon mucosa

**DOI:** 10.1016/j.ejphar.2023.175897

**Published:** 2023-09-05

**Authors:** Caryl Evans, Kathryn Howells, Rie Suzuki, Alastair J.H. Brown, Helen M. Cox

**Affiliations:** a King's College London, Wolfson Centre for Age-Related Diseases, Institute of Psychology, Psychiatry and Neuroscience, Hodgkin Building, Guy's Campus, London, SE1 1UL, UK; b Heptares Therapeutics Ltd, Steinmetz Building, Granta Park, Great Abington, Cambridge, CB21 6DG, UK; c Northern General Hospital, Herries Road, Sheffield, S5 7AU, UK

**Keywords:** TRPV1, TRPA1, Mucosal ion transport, Gastrointestinal regional specificity

## Abstract

Capsaicin and allyl isothiocyanate (AITC) activate transient receptor potential (TRP) vanilloid-1 (TRPV1) and TRP ankyrin-1 (TRPA1), respectively. TRPV1 and TRPA1 expression have been identified in the gastrointestinal (GI) tract. GI mucosal functions remain largely undefined for TRPV1 and TRPA1 with side-dependence and regional differences in signalling unclear. Here we investigated TRPV1- and TRPA1-induced vectorial ion transport as changes in short-circuit current (ΔI_sc_), in defined segments of mouse colon mucosa (ascending, transverse and descending) under voltage-clamp conditions in Ussing chambers. Drugs were applied basolaterally (bl) or apically (ap). Capsaicin responses were biphasic, with primary secretory and secondary anti-secretory phases, observed with bl application only, which predominated in descending colon. AITC responses were monophasic and secretory, with ΔI_sc_ dependent on colonic region (ascending vs. descending) and sidedness (bl vs. ap). Aprepitant (neurokinin-1 (NK1) antagonist, bl) and tetrodotoxin (Na^+^ channel blocker, bl) significantly inhibited capsaicin primary responses in descending colon, while GW627368 (EP4 receptor antagonist, bl) and piroxicam (cyclooxygenase inhibitor, bl) inhibited AITC responses in ascending and descending colonic mucosae. Antagonism of the calcitonin gene-related peptide (CGRP) receptor had no effect on mucosal TRPV1 signalling, while tetrodotoxin and antagonists of the 5-hydroxytryptamine-3 and 4 receptors, CGRP receptor, and EP1/2/3 receptors had no effect on mucosal TRPA1 signalling. Our data demonstrates the regional-specificity and side-dependence of colonic TRPV1 and TRPA1 signalling, with involvement of submucosal neurons and mediation by epithelial NK1 receptor activation for TRPV1, and endogenous prostaglandins and EP4 receptor activation for TRPA1 mucosal responses.

## Highlights

• Neurokinins mediate the initial phase of TRPV1-induced epithelial ion secretion.
• TRPV1 signalling is restricted to the basolateral side of the descending colonic region.
• TRPA1-induced ion secretion is prostaglandin and EP4 receptor-mediated.
• Colonic TRP channel signalling shows region- and side-dependence.

## 1 Introduction

Transient receptor potential (TRP) vanilloid-1 (TRPV1) and TRP ankyrin-1 (TRPA1) are ligand-gated non-selective cation channels. TRPV1 is activated by capsaicin (Tominaga et al., 1998), noxious heat (Caterina et al., 1997), noxious chemicals, including protons (Petersen and LaMotte, 1993) and peptide toxins (Siemens et al., 2006; Bohlen et al., 2010). TRPA1 in contrast is activated by noxious cold temperature (Story et al., 2003), mechanical stimuli (Kwan et al., 2006) and pungent compounds for example, allyl isothiocyanate (AITC) (Jordt et al., 2004).

TRPV1 and TRPA1 are expressed on small-diameter unmyelinated C-type and thinly myelinated A***δ***-type fibres of the sensory ganglia, including dorsal root ganglia (DRG), trigeminal ganglia and nodose ganglia (NG) (Caterina et al., 1997; Story et al., 2003; Nagata et al., 2005). Within the gastrointestinal (GI) tract, TRPV1 and TRPA1 expression has been identified in the stomach, small intestine and colon of the rat, mouse and human, the major source originating from gut-projecting neurons of the NG and DRG (Tan et al., 2008; Holzer, 2011). Anterograde tracing of DRG to the mouse colon has shown spinal afferent terminals to be almost exclusively calcitonin gene-related peptide (CGRP) immunoreactive (Spencer et al., 2014) and these fibres are >90% immunoreactive to TRPV1 (Sharrad et al., 2015). The presence of TRPV1 in the enteric nervous system (ENS) however is controversial. Early studies suggest TRPV1 is present on intrinsic primary afferent neurons (IPANs), as well as on epithelial cells of the gastric and small intestinal mucosae (Anavi-Goffer et al., 2002; Kato et al., 2003; Faussone-Pellegrini et al., 2005; Holzer, 2011). However, numerous other studies have failed to identify TRPV1-immunoreactivity in enteric neurons (Patterson et al., 2003; Ward et al., 2003; Horie et al., 2004; Sharrad et al., 2015). On the other hand, TRPA1 has been identified on inhibitory motor neurons, descending interneurons, cholinergic neurons, and IPANs of the ENS (Poole et al., 2011) and on non-neuronal cells, for example, 5-hydroxytryptamine (5-HT)-secreting enterochromaffin (EC) cells and cholecystokinin-secreting enteroendocrine cells of the human and rodent GI mucosae (Purhonen et al., 2008; Nozawa et al., 2009).

In the GI tract, TRPV1 and TRPA1 modulate local functions such as mucosal anion secretion (Yarrow et al., 1991; Kaji et al., 2012; Fothergill et al., 2016), motor activity (Penuelas et al., 2007; Matsumoto et al., 2009; Nozawa et al., 2009) and vascular perfusion (Kono et al., 2013). Commonly, these functions are achieved via the release of neurotransmitters/peptides from afferent nerve endings which can act directly, stimulating different target cells e.g., epithelia and smooth muscle cells (de Man et al., 2008; Yarrow et al., 1991), or indirectly, through a neurogenic process, identified through atropine- and/or TTX-sensitivity, involving submucosal neurons (primarily involved in mucosal secretion) (Vanner and MacNaughton, 1995) and myenteric neurons (predominantly involved in GI motility) (Someya et al., 2003; Penuelas et al., 2007).

Of importance is the heterogeneity and regional differences in the receptors/channels expressed by neurons and EC cells (as well as the associated neuropeptide/transmitter contents) along the length of the GI tract (Martin et al., 2017; Drokhlyansky et al., 2020; Koo et al., 2021; Burclaff et al., 2022). For example, we have observed that mucosal responses to glucagon-like peptide 1 and CGRP showed significant intracolonic variability in mouse (Tough et al., 2018). Therefore, the signalling mechanisms following TRPV1 and TRPA1 activation are also likely to be region- (and species)-dependent. Furthermore, with functional evidence inferring both luminal and serosal TRPA1 activity it is also important to consider potential signalling sidedness in the mucosae. Most studies to date overlook these aspects, limiting our understanding of potential region- and/or side-dependence in TRP channel-mediated physiological functions along the colonic length, thus forming the main aims of this study.

## 2 Materials and methods

### 2.1 Materials

AITC and (1E,3E)-1-(4-Fluorophenyl)-2-methyl-1-penten-3-one oxime (A967079) were gifted by Professor S. Bevan and Dr D. Andersson. Aprepitant and 6-Isopropoxy-9-xanthone-2-carboxylic acid (AH 6809) were purchased from Bio-Techne (Abingdon, UK), 1-[3,5-Dibromo-*N*-[[4-(1,4-dihydro-2-oxo-3(2*H*)-quinazolinyl)-1-piperidinyl]carbonyl]-D-tyrosyl-L-lysyl]-4-(4-pyridinyl)-piperazine (BIBN4096), capsazepine and tetrodotoxin (TTX) from Tocris (Bristol, UK), substance P and rat αCGRP from Bachem (St Helens, UK), 4-(4-,9-Diethoxy-1,3-dihydro-1-oxo-2*H*-benz[f]isoindol-2-yl)-*N*-(phenylsulfonyl) benzeneacetamide (GW627368) from Generon (Slough, UK) and prostaglandin E_2_ (PGE_2_) from Santa Cruz (Dallas, USA). Piroxicam, tropisetron, 1-[4-Amino-5-chloro-2-(3,5-dimethoxy-phenyl)methyloxy]-3-[1-[2-methylsulphonylamino]ethyl]piperidin-4-yl]propan-1-one hydrochloride (RS39604), 5-HT, 5-bromo-*N*-(4,5-dihydro-1*H*-imidazole-2-yl)-6-quinoxalinamine (UK 14,304) and capsaicin were all purchased from Sigma (Poole, UK). Forskolin were obtained from Abcam (Cambridge, UK). All small molecules were dissolved in neat dimethyl sulfoxide (DMSO, at 1 mM), capsaicin and capsazepine were dissolved in ethanol (EtOH; 95%) and peptides and toxin were dissolved in distilled water (dH_2_O). All stock aliquots were stored at -20°C until required and underwent one freeze-thaw cycle only, apart from AITC which was stored at +4°C.

### 2.2 Mucosal preparation and measurement of changes in vectorial ion transport as short-circuit current (I_sc_)

Mice (male and female C57BL/6J, 10+ weeks old, procured from Charles River Laboratories (Margate, UK)) had free access to standard chow (Rat and Mouse No.3 Breeding diet; Special Diets Services, Braintree, UK) and water *ad libitum* and were housed in 12 h light-dark cycles, with regulated temperature (22 ± 2°C) and humidity (55% ± 10%) settings. All animal care and experimentation was performed in compliance with the Animals (Scientific Procedures) Act 1986 and were approved by the UK Home Office (licence number: P6EA0199C). Mice were killed by cervical dislocation and the colon excised and submerged in fresh Krebs-Henseleit (KH; in mM; NaCl 118, KCl 4.7, NaHCO_3_ 25, KH_2_PO_4_ 1.2, MgSO_4_ 1.2, CaCl_2_ 2.5, and D-glucose 11.1). The colon was then cut along the mesenteric line and luminal contents removed. The tissue was pinned mucosal side down and the overlying smooth muscle and associated myenteric innervation was removed by blunt microdissection. The mucosa with intact submucosal innervation was segmented, generating 8 adjacent preparations designated as ascending colon (closest to the caecum; AC1-AC3), transverse colon (ATC and TDC) or descending colon (DC3-DC1; closest to the rectum) as described previously (Tough et al., 2018). Each preparation was positioned in an Ussing chamber (exposed area, 0.14 cm^2^) bathed on both sides in circulating aerated (95% O_2_/5% CO_2_) KH maintained at 37°C and voltage-clamped at 0 mV, as described previously (Tough et al., 2011). Once stabilisation of the basal short-circuit current (I_sc_; μA.cm^-2^) (20 min) and transepithelial resistance (TER, Ω.cm^2^; measured by delivering +1 mV pulses and applying Ohm’s law) was achieved, agonist or antagonist additions were made to the apical (ap) or basolateral (bl) reservoirs as specified.

### 2.3 Sidedness, selective antagonism and TTX-sensitivity of colonic TRPV1 and TRPA1 signalling

To investigate response sidedness, the TRPV1 agonist, capsaicin (1 μM) or the TRPA1 agonist, AITC (10 μM) was applied bl or ap to 8 adjacent naïve colonic preparations (AC1-DC1 as described in section 2.2) and the changes in I_sc_ recorded over 40 min.

Selective antagonism studies utilised capsazepine (TRPV1 antagonist) or A967079 (TRPA1 antagonist). Capsazepine (3, 10, 30, 100 μM) or vehicle (0.1% EtOH) were applied bl (only) to 5 adjacent colonic preparations (taken within the TDC-DC1 region) 20 min prior to capsaicin (1 μM; bl) and the maximum changes in I_sc_ were recorded. Alternatively, A967079 (1, 3 or 10 μM) or vehicle (0.1% DMSO) were applied bl to the ascending colon (4 adjacent preparations taken within the AC1-AC3 region) or ap to the descending colon (4 preparations taken within DC3-DC1) 20 min prior to a single bl (ascending colon) or ap (descending colonic region) AITC (10 μM) addition and the changes in I_sc_ recorded for 30 min.

To investigate TTX-sensitivity, the neurotoxin (100 nM; bl) or vehicle (dH_2_O) were applied to 4 adjacent colonic preparations taken from the TDC-DC1 region, or 4 adjacent preparations taken within the AC1-AC3 region 20 min prior to a capsaicin (1 μM; bl, descending region only) or AITC (10 μM; ap) addition, and the changes in I_sc_ recorded.

### 2.4 Characterisation of regional colonic TRPV1 and TRPA1 signalling

Characterisation of TRPV1 signalling in the descending colon (on 4 preparations from the DC3-DC1 region) involved a single optimal pretreatment of the neurokinin-1 (NK1) receptor antagonist aprepitant (10 μM; bl), the CGRP receptor antagonist BIBN4096 (1 μM; bl, Tough et al., 2018), both in combination, or vehicle (0.1% DMSO). The antagonists (and vehicle) were applied 15 min prior to capsaicin (1 μM; bl) addition and the subsequent changes in I_sc_ were recorded.

Characterisation of TRPA1 signalling in both the ascending colon (4 mucosae from within the AC1-AC3 region) and descending colon (4 adjacent preparations from DC3-DC1) involved a single optimal pretreatment with either aprepitant or BIBN4096, as above, the EP4 receptor antagonist GW627368 (10 μM; bl), the EP1/2/3 combined-receptor antagonist AH 6809 (10 μM; bl), the cyclooxygenase (COX) inhibitor piroxicam (5 μM; bl, Fairbrother et al., 2012), or the 5-HT_3_ and 5-HT_4_ receptor antagonists tropisetron (100 nM; bl) and RS39604 (1 μM; bl, Tough et al., 2020) respectively, or vehicle (0.1% DMSO). Again, the inhibitors (or vehicle) were applied to naïve mucosae 15 min prior to a single bl (AC1-AC3 region only) or ap (DC3-DC1 region only) AITC (10 μM) addition and the subsequent changes in I_sc_ recorded for 30 min.

Following TRP channel activation, agonist controls, substance P (30 nM; bl), CGRP (10 nM; bl), 5-HT (1 μM; bl) or PGE_2_ (1 μM; bl) were used to confirm receptor antagonism by the pretreatments. To end the experiments, direct activation of epithelial adenylate cyclase using forskolin (1 μM; ap) and subsequent reduction of forskolin-elevated I_sc_ levels with Gα_i_-coupled α_2_-agonist, UK 14,304 (1 μM; bl) were also used as internal controls.

### 2.5 Data analysis

Changes in I_sc_ to each agonist and antagonist (or vehicle) were pooled and converted to μA.cm^-2^. Pooled I_sc_ responses and TERs are presented throughout as the mean ± standard error of the mean (SEM) of *n* independent experiments (*n* indicates the number of mice used in each study). Data were analysed using GraphPad Prism (version 9.3; GraphPad Prism software, La Jolla, CA). Following normality testing (Shapiro-Wilk test), single comparisons or multiple comparisons between data groups were identified using the unpaired two-tailed Student’s t-test or the one-way analysis of variance (ANOVA) with Dunnett’s or Tukey’s *post hoc* test, respectively. Alternately, when data were not normally distributed single comparisons or multiple comparisons between data groups were identified using the Mann Whitney test or the Kruskal Wallis test, respectively. In all cases *P*<0.05 was considered statistically significant.

## 3 Results

### 3.1 Basal electrophysiological parameters of the ascending, transverse and descending colonic mucosae

Mucosal preparations of the ascending, transverse and descending colon were compared for their basal I_sc_ and TER values, measured following tissue stabilisation and prior to drug additions (Table S1). Both basal I_sc_ and TER values were characteristic of the region, with some regional comparisons displaying significant differences (Table S1). Male and female mice were used throughout this study and neither the basal I_sc_ nor TER, nor the antagonist/agonist-induced changes in I_sc_ showed sex-dependent differences (Fig. S2; as an example).

### 3.2 TRPV1 signalling is more robust on the serosal side of the descending colon

**Fig. 1:**
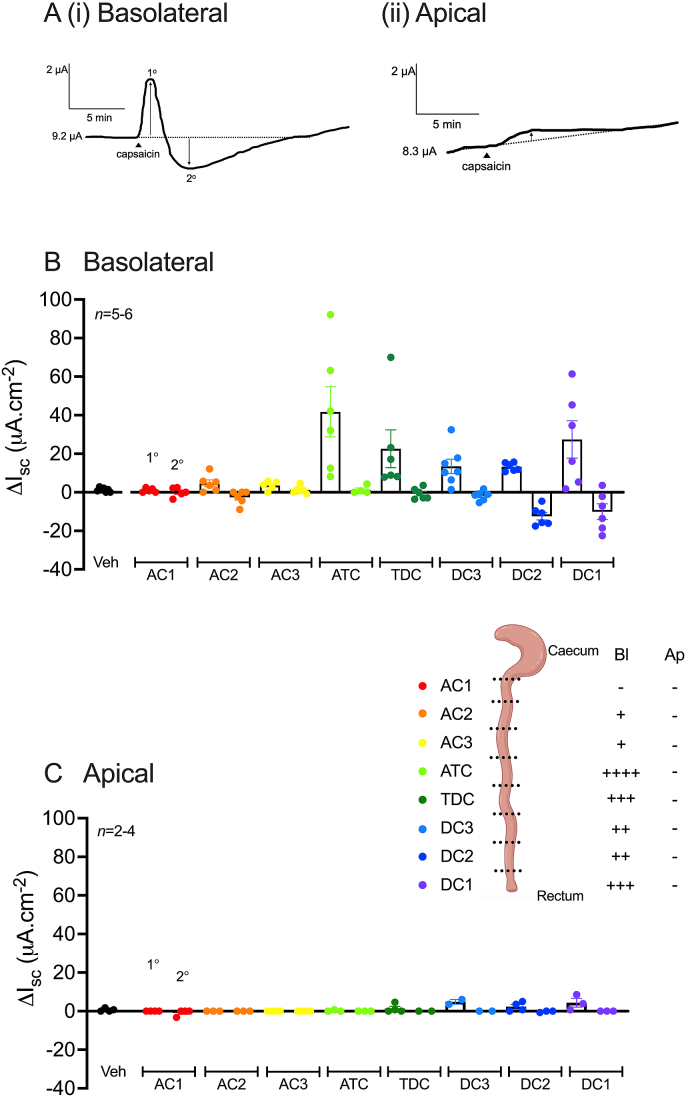
**Basolateral capsaicin elicits a biphasic response along the length of the mouse colon, which is greater in the distal regions.** (A) Representative traces of the capsaicin response following bl (i) or ap (ii) addition recorded from the DC2 colonic region. Capsaicin (1 μM) or vehicle (0.1% EtOH) were applied to naïve mucosae bl (B) or ap (C) and the maximum changes in I_sc_ recorded. Bars represent mean ±1SEM, from n numbers shown and are colour-coded according to the schematic of colon regions (created with BioRender.com).

To establish whether TRPV1 signalling shows regional variation and sidedness the TRPV1 agonist capsaicin (1 μM; Fig. S1A) was applied bl or ap to mucosae from the most ascending colon (AC1) to the most descending region (DC1). Bl capsaicin elicited a rapid biphasic change in I_sc_; a transient primary (1^o^) increase in I_sc_ reaching a peak at 1-3 min, followed immediately by a slower secondary (2^o^) decrease in I_sc_ reaching its nadir between 5 and 10 min (Fig. 1A(i)). The bl capsaicin response (Fig. 1B) differed along the colonic length, with greater 1^o^ responses observed in the transverse region (ATC and TDC), while in the descending region (DC3-DC1) greater 1^o^ increases and 2^o^ decreases in I_sc_ were observed. Neither phase of this response was as robust in the ascending colon (AC1-AC3) (Fig. 1B). In contrast, the ap capsaicin response (Fig. 1A(ii) and 1C) was negligible along the entire colonic length (AC1-DC1). This, in conjunction with the small bl capsaicin responses observed in the ascending colon limited subsequent TRPV1 signalling characterisation (sections 3, 3.3.4) to the transverse and descending colon (TDC-DC1) with bl capsaicin administration only. Internal controls, forskolin and UK14,304 mediated consistent increases or decreases in I_sc_ respectively, confirming the expected mucosal responses (data not shown).

### 3.3 The effects of capsazepine or TTX on the capsaicin response

**Fig. 2:**
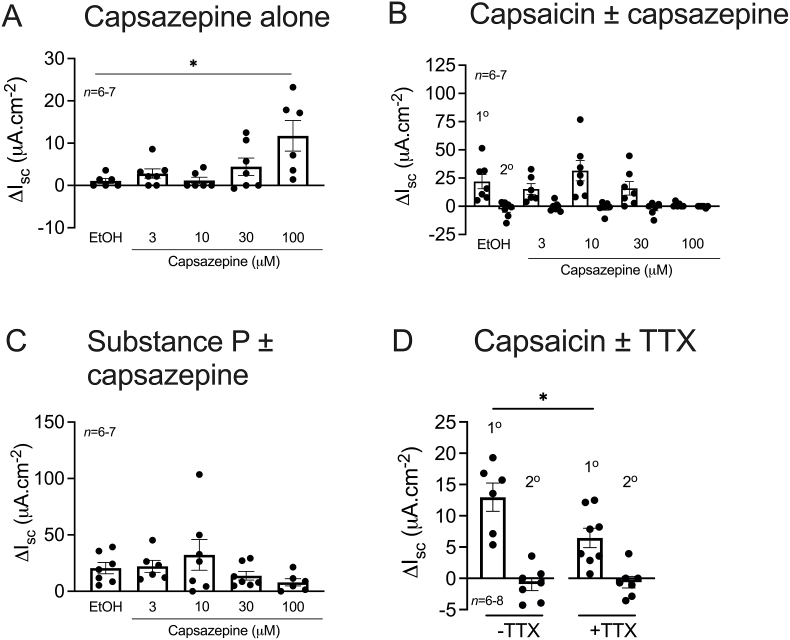
**TRPV1 antagonism with capsazepine (100 μM) or TTX blunts capsaicin signalling in mouse descending colon.** Capsaicin (1 μM) was applied to colonic mucosae bl following pretreatment with increasing concentrations of capsazepine (3, 10, 30, 100 μM) or vehicle (0.1% EtOH) (A) and the biphasic capsaicin response recorded (in I_sc_) (B). Substance P (30 nM, C) applied after capsaicin was used as the internal control. Alternatively, capsaicin (1 μM; bl) was applied to colonic mucosae after a TTX (100 nM; bl) pretreatment and the biphasic change in I_sc_ recorded (D). Data bars represent mean ±1SEM, from n numbers shown. *P<0.05 (Kruskal-Wallis test, A and Student’s t-test, D).

TRPV1 antagonist capsazepine (3, 10, 30, 100 μM) or vehicle (0.1% EtOH) was applied bl to 5 adjacent colonic preparations (taken within the TDC-DC1 region) 20 min prior to a single addition of capsaicin (1 μM; bl). Capsazepine at 100 μM (only) significantly elevated I_sc_ revealing channel-mediated tonic inhibitory activity in the descending colon (Fig. 2A). Capsazepine at 100 μM abolished the primary phase of the capsaicin response (Fig. 2B) however this was not statistically significant. Following activation of TRPV1 on neurons, substance P is released from nerve terminals and in turn activates epithelial expressed (Gα_q_-coupled) NK1 receptors inducing ion secretion (as investigated in section 3.4). To confirm the specificity of capsazepine for TRPV1, substance P (30 nM; bl), was used to ensure downstream signalling components were unaffected. Capsazepine had no significant effect on subsequent substance P responses (Fig. 2C). TTX (100 nM) reduced the descending colonic mucosae basal I_sc_ (-3.1 ± 1.8 μA cm^-2^ (*n* = 7) cf. vehicle (dH_2_O) 0.0 ± 0.0 μA cm^-2^ (*n* = 7)) and significantly inhibited the primary phase of the capsaicin response, while having no effect on the secondary phase (Fig. 2D).

### 3.4 Interrogation of neurokinins and CGRP in TRPV1-mediated signalling in the descending colon

**Fig. 3:**
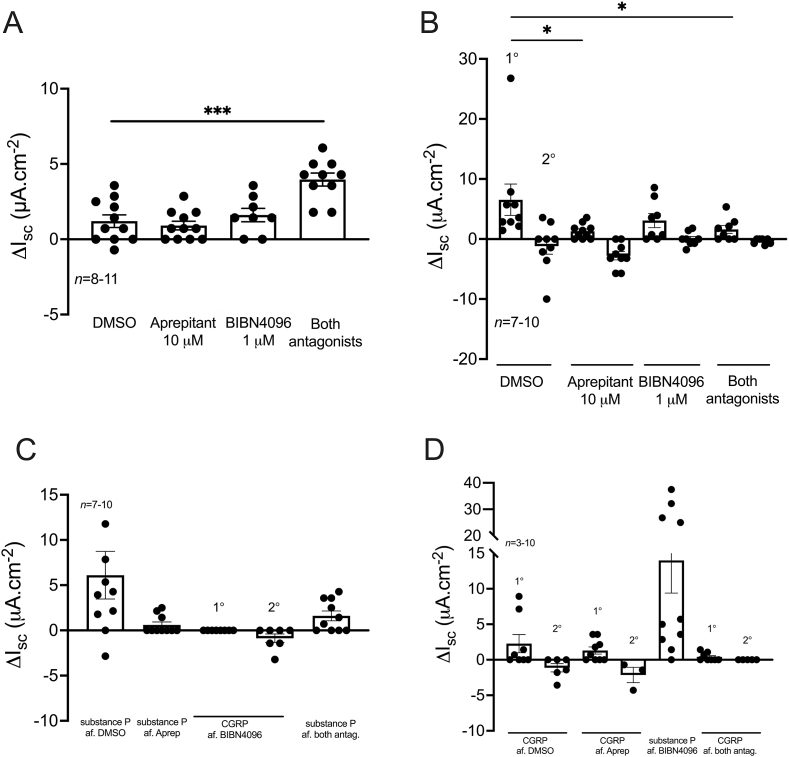
**The primary phase of the capsaicin response is mediated by NK1 receptors in the mouse descending colon.** Descending colonic mucosae were pretreated bl with vehicle (0.1% DMSO), aprepitant, BIBN4096, or both antagonists combined (A). Capsaicin (1 μM; bl) was then applied and the maximum changes in I_sc_ recorded (B). Internal controls (C&D) were applied at the end of the experiment, with substance P (30 nM; bl) applied prior to CGRP (10 nM; bl) if aprepitant (or DMSO, or both antagonists in combination) was the pretreatment used. Alternatively, CGRP was applied prior to substance P if BIBN4096 was the pretreatment. Bars represent the mean ±1SEM, from n numbers shown. Statistical difference between vehicle and antagonist responses was *P<0.05; ***P<0.001 (one-way ANOVA with Dunnett’s post hoc test (A) and Kruskal-Wallis test (B)).

To establish the endogenous mediators of colonic TRPV1 signalling, mucosal preparations (4 from the most descending region (DC3-DC1)) were pretreated with a single optimised concentration of the NK1 receptor antagonist, aprepitant (10 μM; bl, Fig. S3A), the CGRP receptor antagonist, BIBN4096 (1 μM; bl, Fig. S3B), both antagonists combined, or vehicle (0.1% DMSO). Individually, aprepitant and BIBN4096 had no effect on descending colonic secretory tone when compared to vehicle (Fig. 3A). However, the NK1 and CGRP antagonists in combination, significantly increased I_sc_ *per se* revealing a degree of combined endogenous NK1- and CGRP-receptor-mediated tonic inhibitory activity in the descending colon (Fig. 3A). Aprepitant significantly reduced the primary capsaicin-induced increase in I_sc_ (Fig. 3B) while BIBN4096 had no significant inhibitory effect on either aspect of the biphasic capsaicin response (Fig. 3B). Like aprepitant, both antagonists in combination significantly inhibited the primary capsaicin phase (Fig. 3B). Agonist controls, substance P and CGRP confirmed their mucosal responses and the effectiveness of both aprepitant and BIBN4096 at inhibiting NK1 and CGRP receptor signalling respectively (Fig. 3C&D).

As briefly described above, following TRPV1 activation, substance P is released from nerve terminals and in turn activates epithelial expressed NK1 receptors inducing ion secretion. To understand whether the regional variation in TRPV1 signalling along the colonic length (Fig. 1B) could be due to differences in downstream NK1 receptor activation, the agonist [Sar^9^, Met(O_2_)^11^] substance P (SMSP) was applied bl to naïve colonic mucosae (AC1-DC1). There was no apparent regional difference in NK1 receptor signalling along the length of the mouse colon (Fig. S4).

In summary, capsaicin responses are biphasic, bl restricted and they predominate in the descending colon. Capsaicin-induced activation of TRPV1 specifically was confirmed using selective antagonist capsazepine. The primary phase of the capsaicin response was both TTX sensitive and NK1 receptor mediated, whereas the mediators of the secondary phase remain unclear. Finally, the regional differences in capsaicin signalling appear to be TRPV1-dependent rather than occurring through downstream mechanisms e.g., NK1 receptor activation, which did not show significant region-dependent variation.

### 3.5 TRPA1 signalling shows colonic regional and mucosal surface preference

**Fig. 4:**
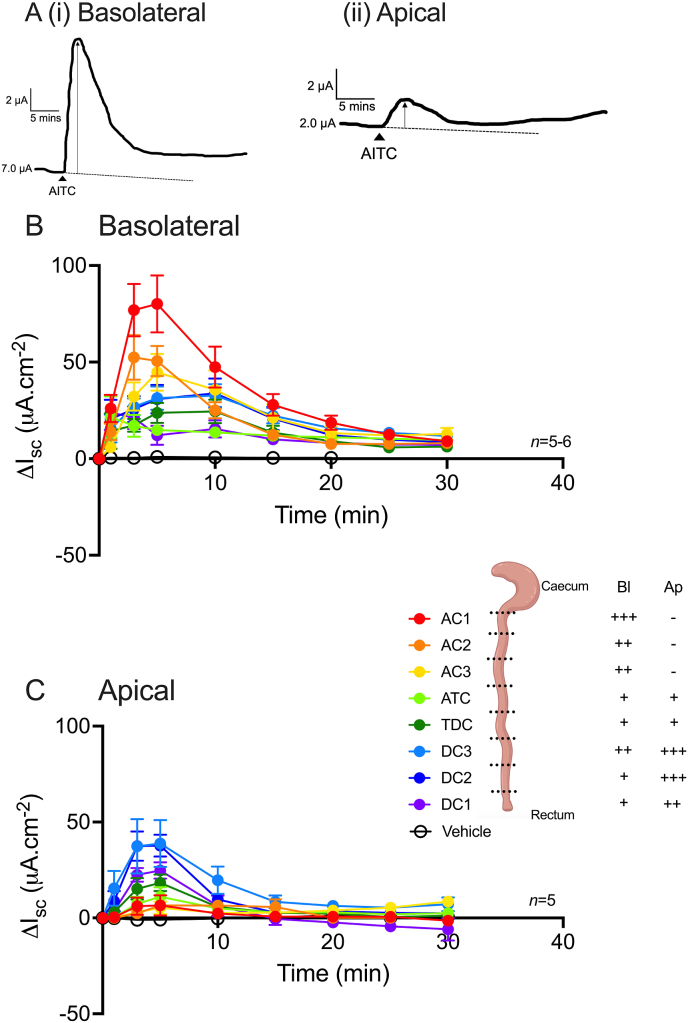
**The AITC response maxima changes along the length of the mouse colon dependent on the side of application.** (A) Representative traces of the AITC response following bl (A (i)) or ap (A (ii)) addition recorded from the AC1 region. AITC (10 μM) or vehicle (0.1% DMSO) were applied to naïve mucosae either bl (B) or ap (C) and the change in I_sc_ recorded over 30 min. Data points represent the mean ±1SEM, from n numbers shown, and are colour-coded according to the schematic of colon regions (created with BioRender.com).

To establish whether TRPA1 signalling shows regional variation and sidedness the TRPA1 agonist AITC (10 μM; Fig. S1B), was applied bl (Fig. 4B) or ap (Fig. 4C) along the length of the naïve colon, from the most ascending (AC1) to the most descending (DC1) mucosal preparation. AITC administered to either side initiated monophasic increases in I_sc_ which peaked within 10 min (Fig. 4A (i) and (ii) show example traces from the AC1 region). Upon bl AITC application the greatest increases in I_sc_ were observed in the ascending colonic regions (specifically AC1), while the bl AITC responses observed in the transverse colon (ATC and TDC) and most descending colonic region (DC1) were much smaller showing an 81.6%, 70.3% and 82.8% reduction in AITC response respectively compared to AC1 at the 5 min timepoint (Fig. 4B). Interestingly, the opposite response profile was observed following ap AITC addition. The greatest ap AITC response was observed in the descending colon (specifically DC3), while the smallest changes in I_sc_ induced by ap AITC were observed in the ascending colonic regions (AC1-AC3), with an 83.1%, 83.1% and 86.4% reduction in AITC response respectively compared to DC3 at the 5 min timepoint (Fig. 4C).

### 3.6 Selective TRPA1 antagonism inhibits the AITC response, while TTX has no effect

**Fig. 5:**
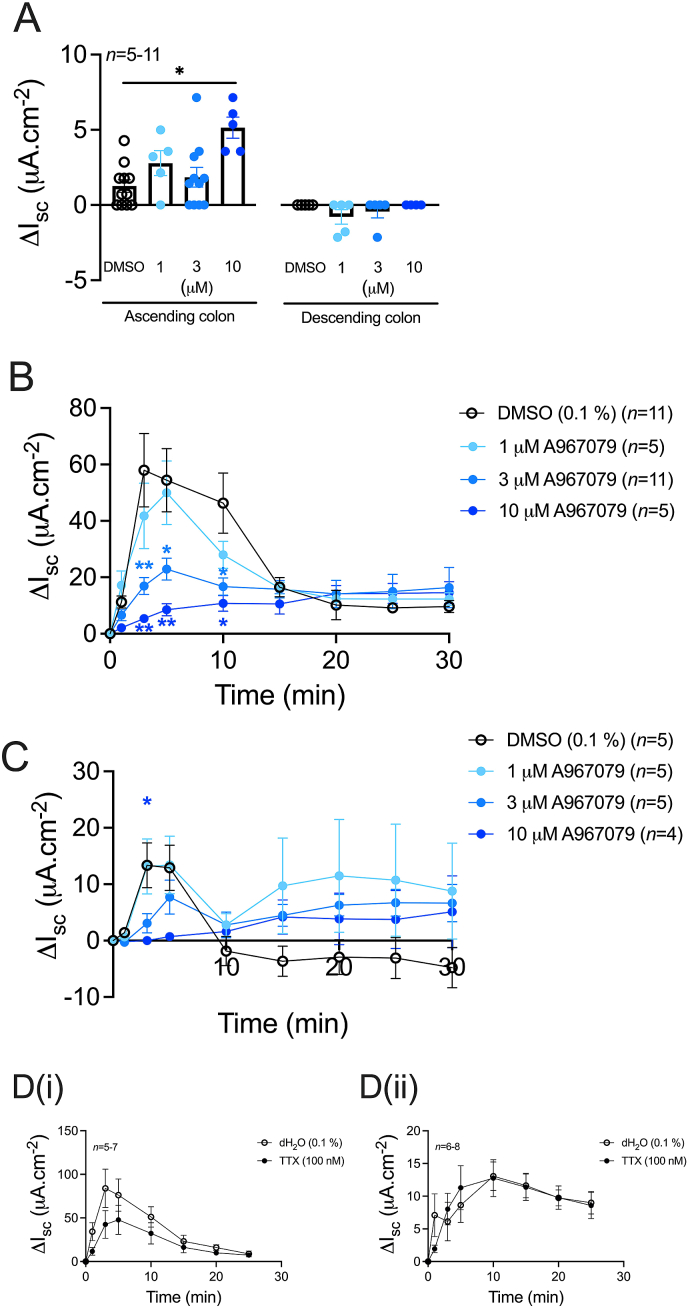
**AITC responses in the mouse colon are inhibited by TRPA1 antagonism and are unaffected by TTX.** Mucosae were pretreated with single increasing concentrations of the TRPA1 antagonist A967079 (1, 3 or 10 μM; bl in ascending colonic mucosae and ap in descending colonic mucosae) or vehicle (0.1% DMSO) (A). AITC (10 μM) was then applied to the ascending colonic mucosae (B) or descending colonic mucosae (C) (administered to the same side as the pretreatments) and the changes in I_sc_ recorded. Alternatively, AITC (10 μM; bl) was applied to ascending (D(i)) or descending (D(ii)) colonic mucosae after TTX (100 nM; bl) pretreatment and the change in I_sc_ recorded over 25 min. Data points represent mean ±1SEM, from n numbers shown. Statistical differences between responses following vehicle or antagonist pretreatment were *P<0.05, **P<0.01 (Kruskal-Wallis test (A and C) and one-way ANOVA with Dunnett’s post hoc test (B)).

To establish AITC-TRPA1 selectivity, ascending and descending colonic mucosae were pretreated with single increasing concentrations of the selective TRPA1 antagonist A967079 (bl application in the ascending colon and ap application in the descending colon) prior to AITC (10 μM) application (i.e. AITC was added to the same side as the pretreatments in each region). A967079 at 10 μM (only) caused a small but significant increase in basal I_sc_ in the ascending colon, revealing a degree of channel-mediated tonic inhibitory activity in this particular region (Fig. 5A). Increasing concentrations of A967079 induced a concentration-dependent reduction in the AITC response in both the ascending and descending colon (Fig. 5 B&C respectively). The maximum change in I_sc_ to AITC in the ascending colon (between 3 and 10 min; Fig. 5B) was significantly reduced following 3 or 10 μM A967079 (when compared to the vehicle response), while in the descending colon the maximum change in I_sc_ (between 3 and 5 min; Fig. 5C) was significantly reduced following 10 μM A967079 only. TTX (100 nM) reduced the ascending (significantly) and descending colonic mucosae basal I_sc_ (-4.1 ± 1.6 μA cm^-2^ (*n* = 7) and -0.63 ± 0.7 μA cm^-2^ (*n* = 8), respectively (Mann-Whitney test)) when compared to vehicle (dH_2_O, 0.0 ± 0.0 μA cm^-2^ (*n* = 7-8)), while having no significant effect on bl AITC responses in both the ascending (Fig. 5D(i)) and descending (Fig. 5D(ii)) colon.

### 3.7 TRPA1 signalling in the ascending and descending colon is mediated by endogenous prostaglandins and subsequent EP4 receptor activation

In order to establish a potential dual neuro-epithelial mediation of colonic TRPA1 signalling, mucosal preparations (4 from the most ascending region (AC1-AC3) and 4 from the most descending region (DC3-DC1)) were pretreated with vehicle (0.1% DMSO) or a single blocking concentration of the NK1 receptor antagonist, aprepitant, the CGRP receptor antagonist, BIBN4096 (as in section 3.4), the 5-HT_3_ and 5-HT_4_ receptor antagonists tropisetron (100 nM; bl) and RS39604 (1 μM; bl) respectively, the COX inhibitor piroxicam (5 μM; bl), the EP4 receptor antagonist GW627368 (10 μM; bl), or the EP1/2/3 combined-receptor antagonist AH 6809 (10 μM; bl), followed by either a bl (to AC1-AC3) or an ap addition (to DC3-DC1) of AITC (10 μM).

**Fig. 6:**
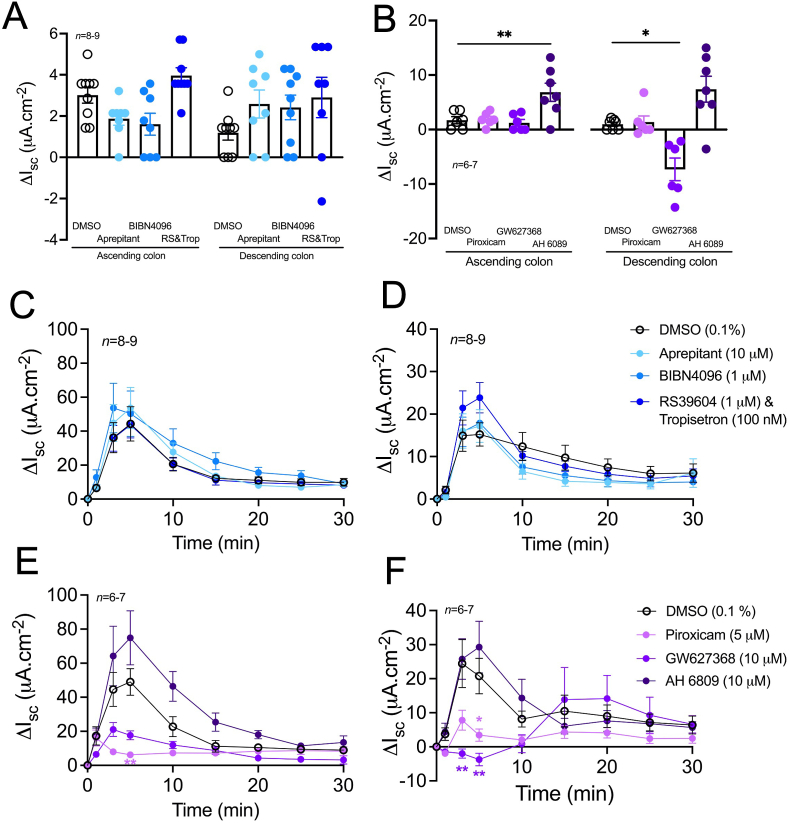
**The AITC response is mediated by endogenous prostaglandins and the EP4 receptor in the mouse colon.** Vehicle (0.1% DMSO), aprepitant, BIBN4096, RS39604 and tropisetron (A) or, piroxicam, GW627368 or AH 6809 (B) were bl applied to the ascending and descending colon mucosae and the effects on basal I_sc_ recorded. AITC (10 μM) was then applied to the ascending colon bl (C&E) and the descending colon ap (D&F) and the change in I_sc_ recorded over 30 min. Data points represent the mean ±1SEM, from n numbers shown. Statistical differences between responses induced by, or following vehicle or antagonist pretreatments were *P<0.05, **P<0.01 (Kruskal-Wallis test (B) or one-way ANOVA with Dunnett’s post hoc test (E&F)).

Neither aprepitant, BIBN4096 nor RS39604 and tropisetron had a significant effect on basal I_sc_ levels in either colonic region (Fig. 6A). Furthermore, neither aprepitant, BIBN4096 nor RS39604 and tropisetron altered the AITC response in the ascending (Fig. 6C) or descending (Fig. 6D) colon, but they did block subsequent NK1, CGRP and 5-HT responses, respectively in both colonic regions (Fig. S5A). In contrast, AH 6809 increased basal I_sc_ in the ascending (significantly) and descending colon, suggesting that collectively EP1/2/3 receptors mediate a degree of anti-secretory tone in the colon (Fig. 6B). In contrast, GW627368 significantly decreased basal I_sc_ in the descending colon (only) inferring that tonic EP4 receptor activity mediates epithelial anion secretion in the descending colon mucosa (Fig. 6B). AH 6809 had no significant effect on the AITC response in the ascending or descending colon (Fig. 6 E&F). Piroxicam, in the ascending and descending colon, and GW627368, in the descending colon, significantly inhibited the AITC response at 3 and/or 5 min (Fig. 6 E&F). The latter part of the AITC response however was not affected by piroxicam or GW627368 with the TRPA1 responses almost overlapping from 15 min onwards (Fig. 6 E&F). Of note, PGE_2_ responses were significantly greater in the ascending colon compared with those in the descending colon and were consistently blocked following EP4 receptor antagonism (Fig. S5B).

To summarise, AITC responses are monophasic and show both side- (ap vs. bl) and region-dependence (ascending vs. descending). AITC-induced activation of TRPA1 specifically was confirmed using selective antagonist A967079. The AITC response was TTX resistant and mediated via endogenous prostaglandin synthesis and subsequent EP4 receptor activation in both colonic regions.

## 4 Discussion

Colonic functions vary; the ascending colon absorbs the majority of vitamins and electrolytes in indigestible material entering from the caecum. The descending colon in contrast, retains, moves and excretes faeces. Underlying differences in the capacity for (net) Na^+^ and water absorption are greater in the ascending colon than the descending colon (Yau and Makhlouf, 1975; Clauss et al., 1985; Schiller et al., 1988). With these functional differences, we proposed that regional variations and side-dependence of signalling pathways are also likely to vary along the length of the colon. To our knowledge this is the first study to examine TRPV1 and TRPA1 signalling in designated regions of the mouse colon.

### 4.1 TRPV1 signalling is restricted to the serosal side and shows descending colon preference

A robust biphasic (bl) response (consisting of an increase in I_sc_ followed by a decrease in I_sc_) was observed in the descending colon. This biphasic bl TRPV1 response was also observed in rat descending colon (Yarrow et al., 1991) and guinea-pig ileum (Vanner and MacNaughton, 1995). Ap capsaicin caused negligible effects on colonic ion secretion. We are not aware of any studies that describe direct luminal capsaicin responses and presume that the stimulant induces TRPV1-mediated depolarisation of afferent neurons once it translocates to the lamina propria (Kagawa et al., 2003).

TRPV1 expression on IPANs remains controversial, however some studies report TRPV1 colocalisation with IPAN markers in the guinea-pig ileum and colon (Anavi-Goffer et al., 2002; Anavi-Goffer and Coutts, 2003). Submucosal IPANs are intact in our mucosal preparations and are most likely present along the colonic length, as observed for myenteric IPANs (Nestor-Kalinoski et al., 2022) therefore does not explain the regional-dependence in TRPV1 signalling we observed. To date, TRPV1 expression on IPANs of submucosal plexus origin remains undefined and the contribution to TRPV1 signalling from albeit severed extrinsic primary afferent neurons should not be dismissed. TRPV1 of extrinsic origin has been identified in the mucosa of the mouse colon. These studies found greater numbers of TRPV1-positive nerve fibres in the rectum and distal colon compared to the sparse innervation in transverse and proximal colon (Matsumoto et al., 2009, 2011; Engel et al., 2012) and this relative frequency matches the functional sensitivity we observed with bl capsaicin along the colon length.

### 4.2 TRPV1-induced ion secretion is TTX-sensitive and mediated in part by NK1 receptor activation

The primary phase of the bl capsaicin response was NK1 receptor mediated and showed partial TTX-sensitivity. While the mediator(s) of the secondary phase remains unclear. In mouse, the majority of TRPV1-expressing neurons (extrinsic afferents) are substance P- and CGRP-positive (Holzer, 2011; Engel et al., 2012), with mucosal substance P also supplied by IPANs (Holzer and Holzer-Petsche, 1997, 2001) implicating the involvement of these neuropeptides in colonic TRPV1-mediated mucosal ion secretion.

Substance P’s involvement in epithelial ion transport is well established in mouse ileum (Wang et al., 1995), rat jejunum (Crowe et al., 1990), guinea-pig ileum (Kachur et al., 1982) and human colon (Riegler et al., 1999; Moriarty et al., 2001). In mouse ileum, substance P mediates Cl^-^ ion secretion by activation of epithelial Gα_q_-coupled NK1 receptors (Wang et al., 1995). Blunting of capsaicin’s primary phase following NK1 receptor antagonism confirms substance P’s involvement in mouse colonic TRPV1 responses. Vanner and MacNaughton (1995) and Yarrow et al. (1991) both proposed the potential involvement of CGRP in the secondary, anti-secretory phase of the capsaicin response however, the lack of available CGRP receptor-specific antagonists at the time made conclusive elucidation impossible. In mouse, CGRP mediates its post-junctional (anti-)secretory effects by activation of epithelial Gα_s_-coupled CGRP receptors (Cox and Tough, 1994; Tough et al., 2018). Investigation of CGRP’s involvement was complicated by the small reductions in I_sc_ observed in the current study and by the graded changes in the CGRP electrogenic response we have reported in consecutive segments of the mouse colon (Tough et al., 2018). As such, conclusive evidence of CGRP’s involvement in TRPV1 signalling is unclear. In this study, TRPV1 signalling showed a partial, yet significant sensitivity to TTX, suggesting that a proportion of the TRPV1-mediated increase in I_sc_ was dependent on action potential conduction in excitatory submucosal neurons, as seen previously in guinea-pig ileum (Vanner and MacNaughton, 1995), while the TTX-resistant component most likely involves direct activity from TRPV1-epxressing extrinsic nerve terminals, as suggested to be the case in rat descending colon (Yarrow et al., 1991).

### 4.3 TRPA1 signalling shows regional variation and side-dependence

AITC administered to either mucosal surface elicited sustained increases in I_sc_, however, the response size was region- and side-dependent. TRPA1-mediated ion secretion has previously been identified in mouse distal colon (Fothergill et al., 2016), human ascending and sigmoid colon, and rat proximal, middle and distal colon (Kaji et al., 2012) and porcine colon (Manneck et al., 2021; region not specified). Within both the human and rat colon, Kaji et al. (2012) observed side-dependence and regional variation in AITC response maxima, supporting our findings in the mouse colon.

TRPA1-positive innervation of the GI tract is both extrinsic in origin (Brierley et al., 2009) and intrinsic to the ENS (Anand et al., 2008). TRPA1 expression has also been localised to ileal EC cells, with studies proposing TRPA1’s role in luminal chemo-sensation (Bellono et al., 2017), as well as to the surface of epithelial cells in mouse colon (and small intestine) and not to distinct epithelial cell types such as enteroendocrine cells (Poole et al., 2011). These TRPA1 expression patterns indicate a more consistent distribution along the colonic length. Although these studies provide evidence for the presence of TRPA1 on the luminal and serosal mucosal surfaces, they do not explain the regional variation (and associated side-dependence) we observed for TRPA1-mediated ion secretion.

### 4.4 TRPA1 signalling is mediated by endogenous prostaglandins and EP4 receptor activation in both colonic regions

In mouse, most of the extrinsic TRPA1-expressing afferent neurons are TRPV1-, substance P- and CGRP-positive (∼ 97% colocalisation with both TRPV1 and CGRP) (Story et al., 2003; Bautista et al., 2005). Furthermore, TRPA1 is also expressed on IPANs of the myenteric ganglia (Poole et al., 2011) and presumably submucosal IPANs, although as far as we can tell no studies have reported submucosal ganglionic localisation to date. These findings also infer the involvement of neuronally-released substance P and CGRP in TRPA1-mediated mucosal ion transport. However, NK1 and CGRP receptor antagonism had no effect on the AITC response in mouse colon. As the expression of TRPA1 compared to TRPV1, is more heterogenous within the GI tract, this indicates that the different cell types could co-release neuropeptide/transmitter/amine mediators and thus contribute to the differences in mucosal TRP signalling we observed. For example, TRPA1 is expressed by ileal EC cells and induces 5-HT release (Nozawa et al., 2009; Bellono et al., 2017). The released 5-HT could then act via 5-HT_3_ (and possibly 5-HT_4_) receptors on TTX-sensitive excitatory submucosal neurons which in turn evoke an epithelial Cl^-^ secretory response (Cooke et al., 1991; Kellum et al., 1999). However, neither 5-HT_3/4_ receptor antagonism, nor TTX pretreatment altered the colonic AITC response. Together this supports a TRPA1 mucosal mechanism that is direct and epithelial in origin, as seen previously in mouse duodenum and rat, mouse and human colon (Kaji et al., 2012; Fothergill et al., 2016).

Instead, COX inhibition and EP4 receptor antagonism significantly inhibited TRPA1 responses in mucosae from the ascending and descending colon. The involvement of endogenous prostaglandins in TRPA1-mediated ion secretion has previously been identified in mouse distal colon (Fothergill et al., 2016) and porcine colon (Manneck et al., 2021), with subsequent activation of the bl-targeted Gα_s_-coupled EP4 receptors also shown in rat colon (Kaji et al., 2012). TRPA1 expression has been reported on intestinal epithelial cells (Poole et al., 2011) which also express COX-1 and PGE synthase, the enzymes mediating homeostatic prostaglandin synthesis (Cohn et al., 1997; Singer et al., 1998), thus promoting them as potential mediators of TRPA1 signalling. AH 6809, the EP1/2/3 receptor antagonist revealed tonic EP-mediated anti-secretory activity (Fig. 6B; and as observed previously by our group, Fairbrother *et al.*, 2010). As EP3 agonism with sulprostone was the only PGE mechanism that reduced I_sc_ levels (Fairbrother *et al.*, 2010) we therefore infer that inhibition afforded by AH 6809 of this Gα_i_-coupled EP3 response predominates and this results in the increase in basal I_sc_ that we observed. Regional differences in the mediators and their cognate receptors downstream from TRPA1 will most likely contribute to the variation in epithelial AITC response seen along the colon length. This appears to be the case in rat colon where the EP4 receptor, COX-1 and COX-2 enzyme expression increased in isolated crypts prepared from successive descending colonic segments (Kaji et al., 2012). This gradation could also explain why, in the present study, antagonism of the stimulatory Gα_s_-coupled EP4 receptor (with GW627368, Fig. 6B) resulted in a decrease in I_sc_ in the descending colon alone.

## 5 Conclusion

**Fig. 7:**
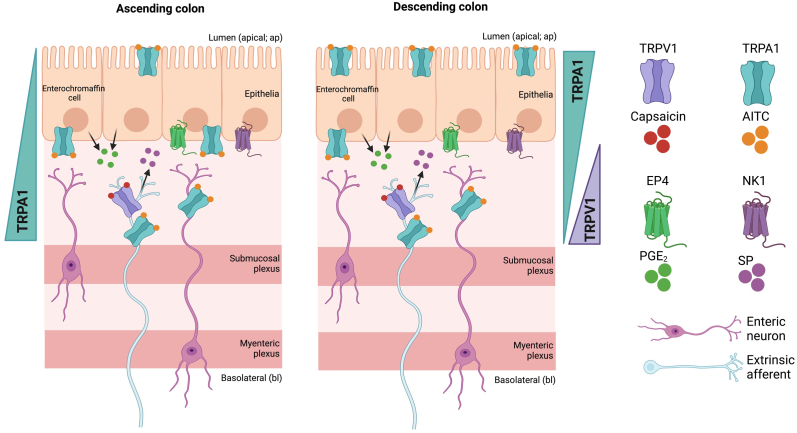
**Simplified schematic highlighting TRPV1 and TRPA1 sidedness and regional specificity and the proposed mucosal signalling mechanisms.** Created with BioRender.com.

TRPV1 signalling is bl, TTX-sensitive and NK1-mediated and more prominent in the transverse-distal colon regions (Fig. 7), contrasting the opposite gradient exhibited by bl TRPA1 signalling which declines down the colonic length and surprisingly switches to the ap surface in the distal half of the mouse colon (Fig. 7). Why acute chemosensation (in this case) changes so radically in colonic mucosae is unclear, but the mediators involved in AITC responses are PGE_2_-EP4-mediated in the large bowel (Fig. 7), in contrast to the 5-HT-dependent mechanism reported for TRPA1 signalling in the EC cells of the upper GI tract (Bellono et al., 2017).

## Credit author statement

Caryl Evans performed the majority of the experiments with contributions from Kathryn Howells. Caryl Evans performed data analyses. Caryl Evans, Rie Suzuki, Alastair J H Brown and Helen M Cox designed the research study and Caryl Evans wrote the paper with input from all the co-authors.

## Declaration of competing interest

The authors have nothing to declare.

## Data Availability

Data will be made available on request.
