# Supplementary material for: Regional characterisation of TRPV1 and TRPA1 signalling in the mouse colon mucosa
Source: Eur J Pharmacol. 2023 Sep 5;954:175897. doi: 10.1016/j.ejphar.2023.175897 (PMC10847397; doi:10.1016/j.ejphar.2023.175897)
Supplement: Multimedia component 1 [file mmc1.docx]

***Supplementary material***

***Table S1. Basal I_sc_ and TER are similar across the length of the murine colon.*** *Values represent mean ± 1SEM from the number of observations shown in parenthesis. Statistical differences in basal I_sc_ and TER between different colonic regions; *P<0.05, **P<0.01, ***P<0.001 (one-way ANOVA with Tukey’s post hoc).*

|  | **AC1** | **AC2** | **AC3** | **ATC** | **TDC** | **DC3** | **DC2** | **DC1** |
| --- | --- | --- | --- | --- | --- | --- | --- | --- |
| **Basal I_sc_ (μA.cm^-2^)** | 36.1 ± 3.3 (16) | 28.9 ± 1.9 (15) | 25.8 ± 2.2 (16) | 33.7 ± 3.0 (16) | 40.6 ± 3.8 (15) | 53.7 ± 3.5 (16) | 40.2 ± 2.1 (15) | 30.3 ± 1.8 (15) |
| **TER**  **(Ω.cm^2^)** | 37.0 ± 3.2 (15) | 41.4 ± 2.9 (16) | 43.9 ± 2.5 (16) | 40.6 ± 1.3 (16) | 33.4 ± 2.4 (16) | 30.0 ± 2.0 (16) | 38.3 ± 3.4 (16) | 51.6 ± 5.1 (16) |

***Basal I_sc_:*** *AC1 vs DC3 ***P<0.001; AC2 vs DC3 ***P<0.001; AC3 vs TDC **P<0.01; AC3 vs DC3 ***P<0.001; AC3 vs DC2 *P<0.05; ATC vs DC3 ***P<0.001; TDC vs DC3 *P<0.05; DC3 vs DC2 *P<0.05; DC3 vs DC1 ***P<0.001*

***TER:*** *AC1 vs DC1 *P<0.05; AC3 vs DC3 *P<0.05; TDC vs DC1 **P<0.01; DC3 vs DC1 ***P<0.001; DC2 vs DC1 *P<0.05*


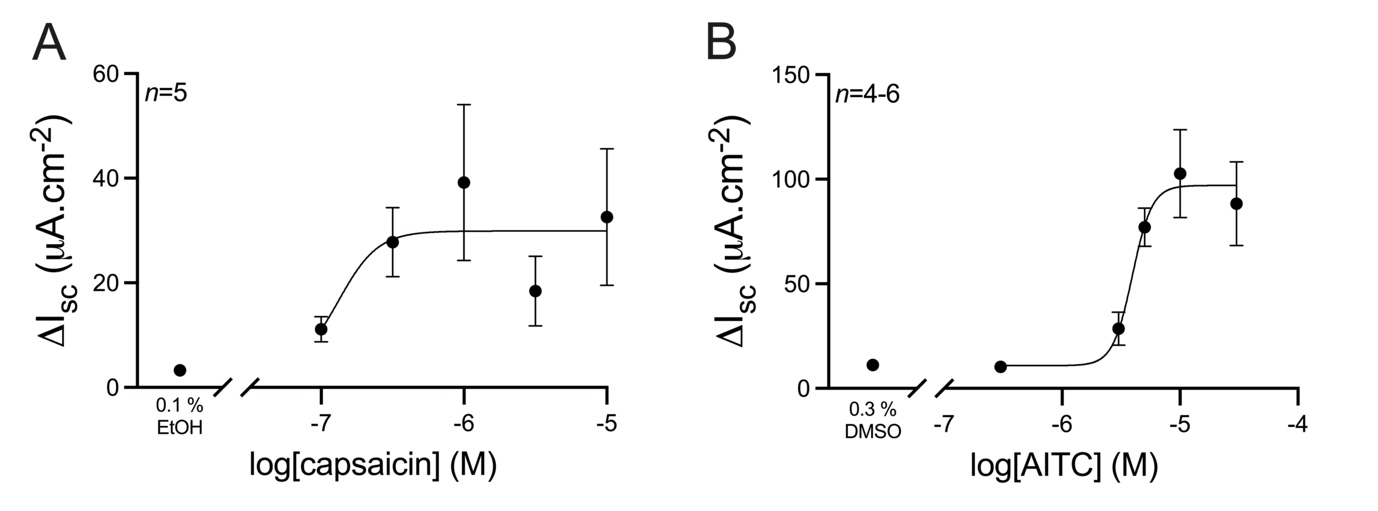


***Figure S1. Capsaicin and AITC concentration-response curves.*** *Single increasing concentrations of capsaicin (A) or AITC (B), or their respective vehicles (EtOH or DMSO) was applied bl to descending or ascending colonic mucosae, respectively and the maximum change in I_sc_ recorded.* *Data* *points* *represent mean ±1SEM, from n numbers shown.*


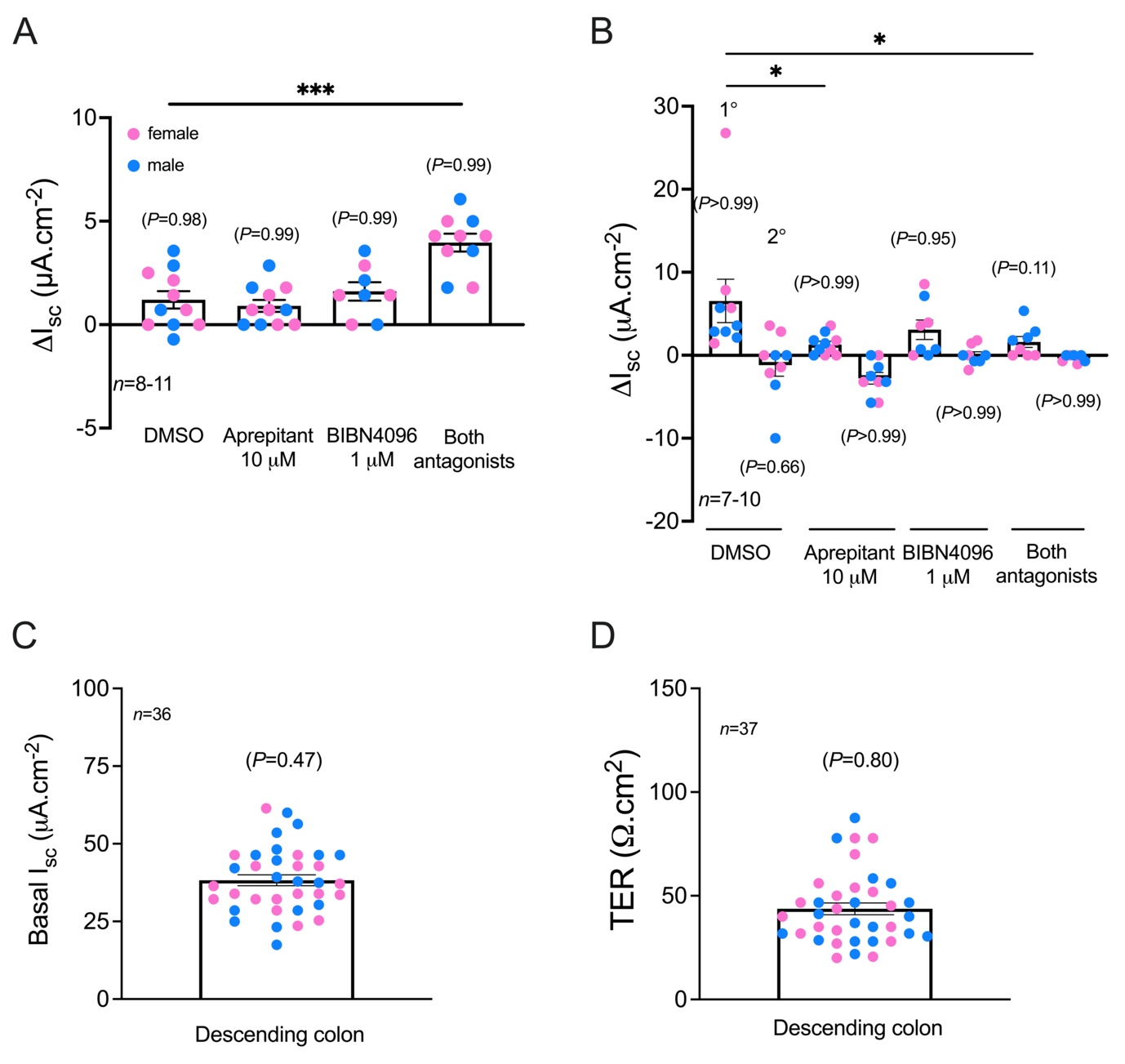


***Figure S2 A-D. There are no sex-dependent differences in antagonist- (A) or capsaicin- (B) induced changes in I_sc_, nor basal I_sc_ (C) and TER (D) readings in descending colonic mucosal preparations.*** *Bars represent mean ±1SEM from n numbers shown. Data points are coloured by sex, pink=female and blue=male. Statistical difference between vehicle and antagonist pretreatments and the following capsaicin responses were *P<0.05; ***P<0.001 (one-way ANOVA with Dunnett’s post hoc (A) and Kruskal-Wallis test (B)). Comparisons between the male and female responses within each data group were performed and the P value shown in parentheses above or below each bar (one-way ANOVA with Šidák’s post hoc (in A), Kruskal-Wallis test (in B), Student’s t-test (in C) and Mann-Whitney test (in D)).*


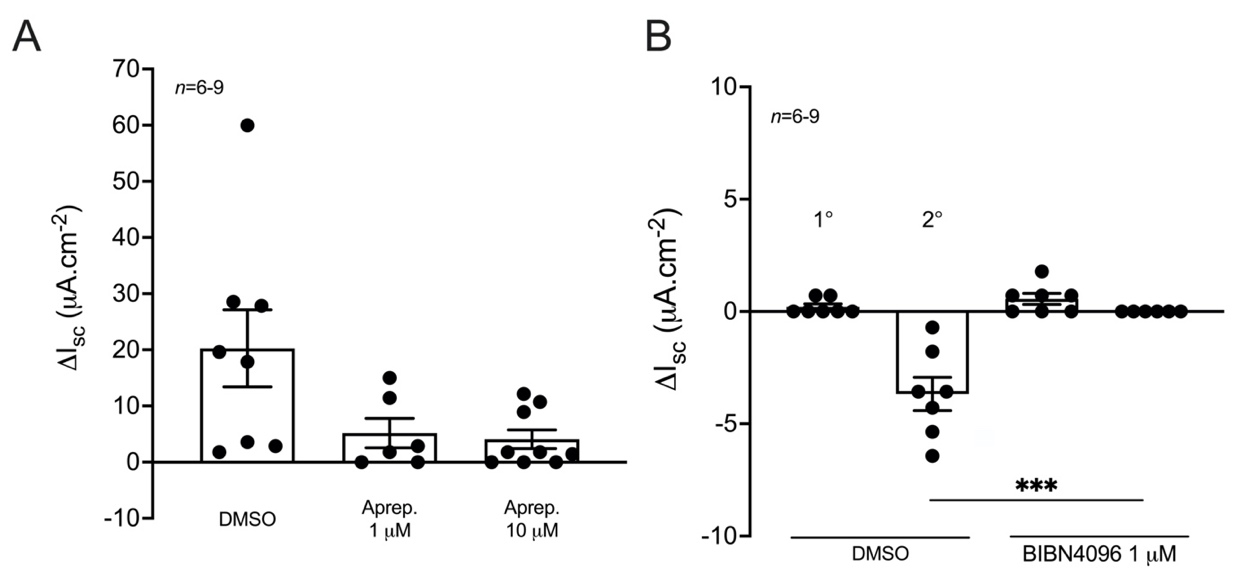


***Figure S3. Aprepitant and BIBN4096 inhibit substance P and CGRP signalling respectively.*** *Substance P (30 nM) (A) or CGRP (10 nM) (B) was applied to descending colonic mucosae bl and the maximum changes in I_sc_ were recorded following pretreatment with either vehicle (0.1 % DMSO), aprepitant or BIBN4069. Bars represent mean ±1SEM, from n numbers shown. Statistical difference between responses following vehicle and antagonist pretreatment was, ***P<0.001 (Student’s t-test).*

*
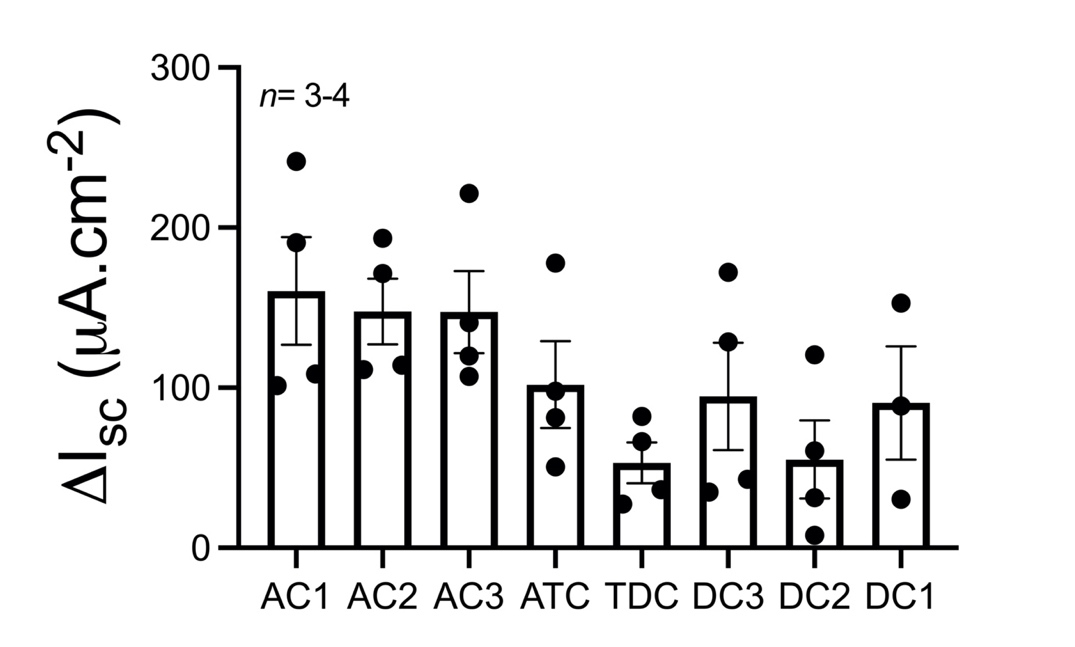
*

***Figure S4. NK1 agonism along the length of the mouse colon.*** *The NK1 receptor agonist* *SMSP (100 nM) was applied to naïve mucosae bl and the maximum change in I_sc_ recorded within 15 min. Bars represent mean ±1SEM, from n numbers shown.*


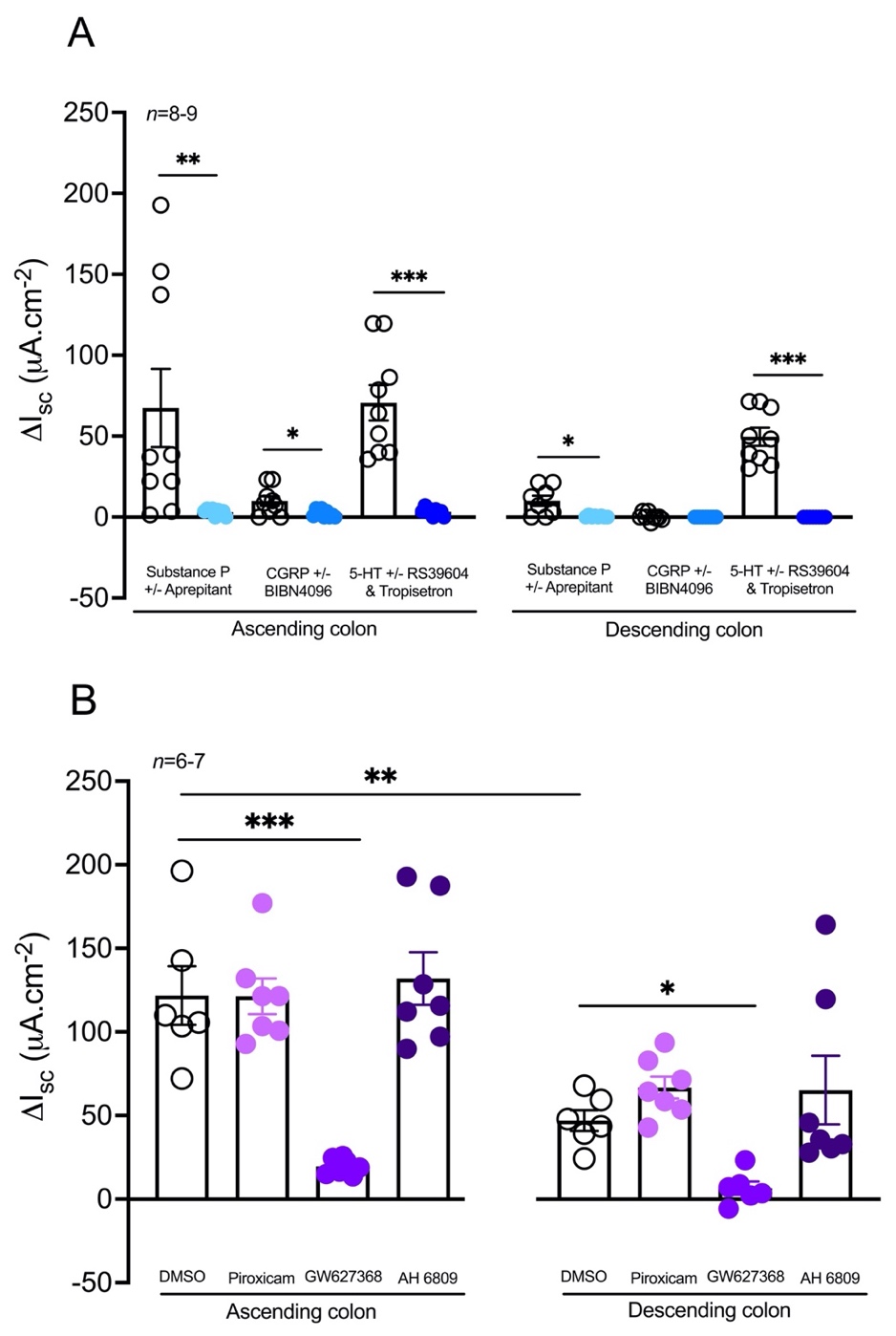


***Figure S5. Substance P, CGRP, 5-HT and PGE_2_ responses were inhibited by aprepitant, BIBN4096, RS39604 and tropisetron and GW627368 respectively in both colonic regions.*** *Substance P (30 nM; (A)), CGRP (10 nM; (A)), 5-HT (1 µM; (A)) or PGE_2_ (1 µM; (B)) was applied to colonic mucosae bl following pretreatment with aprepitant (10 µM), BIBN4096 (1 µM), RS39604 (1 µM) and tropisetron (100 nM), piroxicam (5 µM), GW627368 (10 µM) or AH 6809 (10 µM) or vehicle (DMSO, 0.1 %) and the responses recorded in I_sc_. Data bars represent mean ± 1SEM, from n numbers shown. Statistical differences between responses following vehicle and antagonist pretreatment were *P<0.05, **P<0.01, ***P<0.001 (Student’s t-test, Mann Whitney test or one-way ANOVA with Dunnett’s post hoc).*
